# Comparative analysis of postoperative pain with glycolic acid vs. ethylenediaminetetraacetic acid in endodontic treatment: A randomized clinical trial

**DOI:** 10.4317/jced.62446

**Published:** 2025-04-01

**Authors:** Bruna Tozzati Lago, Doglas Cecchin, Ulysses Lenz, Carlo Theodoro Raymundi Lago, Ana Paula Farina, Matheus Albino Souza, João Paulo de Carli, Yuri Dal Bello

**Affiliations:** 1 Postgraduate Student, Dental School of Universidade de Passo Fundo, Passo Fundo, RS, Brazil; 2 Department of Restorative Dentistry, Dental School of Universidade de Passo Fundo, Passo Fundo, RS, Brazil

## Abstract

**Background:**

The aim of this study was to evaluate pain and analgesic intake after endodontic treatment using glycolic acid (GA) or ethylenediaminetetraacetic acid (EDTA) as the final irrigating solution.

**Material and Methods:**

One hundred fifty patients were randomly assigned to two groups for smear layer removal: 17% GA or 17% EDTA. Postoperative pain was assessed at 24, 48 hours and 7 days. The need for analgesic intake was recorded. Descriptive analysis was performed to assess demographics (Student t, Chi-square and Fisher tests) and study outcomes (Mann Whitney, Friedman and Nemenyi) (*p*<0,05).

**Results:**

Postoperative pain occurred in 52% of the cases. In the 24-hour period, GA had a significantly lower pain score (*p*<0.05). There was no statistical difference between the groups regarding the use of analgesic pills.

**Conclusions:**

GA is associated with less postoperative pain at 24 hours compared to EDTA. No statistically significant difference was observed at 48 hours and 7 days.

** Key words:**Postoperative pain, randomized controlled trial, ethylenediaminetetraacetic acid, glycolic acid, analgesic.

## Introduction

Postoperative pain following endodontic treatment is an acute inflammatory response in the periradicular tissue, occurring in 3% to 58% of patients. Its etiology is multifactorial, involving factors such as pulpal and periapical status, associated signs and symptoms, the use of antibiotics and analgesics, patient age, gender, tooth type and position, the presence of preoperative pain, and periapical lesions. This pain can occur even when root canal procedures are performed carefully and accurately. (1,2).

Final irrigation solutions, such as ethylenediaminetetraacetic acid (EDTA), play a crucial role in removing the smear layer, but they may also be associated with postoperative pain (3,4) as well as cytotoxicity and inter- and peri-tubular erosion (5,6). These factors, in turn, can affect the longevity of the treatment both mechanically and adhesively (7), which drives the search for alternative substances.

Glycolic acid (GA), a small α-hydroxy acid, is derived from organic materials such as sugar cane, beets, and grapes (8). This characteristic is advantageous, especially when compared to EDTA, which generates harmful residues during its manufacturing process (9). GA has been shown to effectively remove the smear layer (10,11) with minimal impact on the mechanical properties of dentin (12). Moreover, the promising cytotoxic and antimicrobial properties of GA (13,14) may reduce periapical tissue injury and alleviate postoperative pain.

Postoperative pain after endodontic treatment is a well-documented condition that has been extensively studied (15,16). Ensuring patient comfort should be a primary focus of research, aiming to improve well-being during and after dental procedures. Therefore, the aim of this study was to evaluate the perception of postoperative pain and analgesic intake in patients undergoing endodontic treatment with different final irrigants. Based on the results of *in vitro* studies on GA, the hypothesis tested is that there is no significant difference in postoperative pain and analgesic intake when compared to EDTA as the final irrigation solution.

## Material and Methods

This double-blind, randomized controlled trial study has been written according to the Preferred Reporting Items for Randomized Trials in Endodontics (PRIRATE 2020 guidelines) (17). This study was approved by the Research Ethics Committee (protocol no. 57417222.6.0000.5342) and registered in the Brazilian Registry of Clinical Trials REBEC (Registro Brasileiro de Ensaios Clínicos, http://www.ensaiosclinicos.gov.br/, database no. RBR-10wz74mt).

-Sample size calculation 

The sample was dimensioned with the aid of the G*power program and a previous study (18). Thus, the minimum sample size that provides a test power of 80% (β=0.20), for an average effect size (d=0.50), with a significance level of 5% (α=0.05), was 56 participants in each group, 112 in total. To compensate for dropout, a total of 156 patients were enrolled for intervention.

Patient selection and randomization

Patients of both genders, aged 18-80 years, were recruited from the pool of patients attending the Endodontic Department from August to December 2022. Healthy patients without chronic diseases (ASA I) and patients with mild systemic diseases (ASA II) were included. Patients were excluded from the study if they had a reported history of allergy to local anesthetics, corticosteroids, or starch; if they had used analgesics or anti-inflammatories within 12 hours before the procedure; if they had used antibiotics within 48 hours before the procedure (to minimize the signs and symptoms of symptomatic apical periodontitis or acute apical abscess); if they were pregnant or lactating; if they were uncontrolled diabetic patients; if they had open apex teeth, root resorption teeth, calcified canals, periodontal mobility (Grade 2 and 3); or if they required antibiotic prophylaxis before treatment.

The final irrigants, 17% GA (pH 2.18) (Natupharma, Rio Grande do Sul, Brazil) and 17% EDTA (pH 7.20) (Biodinamica, Parana, Brazil), were coded as protocol A or B by a pharmacist not involved in the project. Both had vials of the same shape, size, weight, and color. The operator and the subjects had no prior knowledge of which protocol was being used, which is characteristic of a double-blind study. After informed consent was obtained, patients were randomized into two groups by a priori drawing on a randomization website (www.random.org): GA or EDTA as the final irrigant solution.

Anamnesis, clinical examination (palpation, percussion, periodontal probing tests), radiographs, and pulp sensibility tests were performed before the treatment. Patients presenting anterior teeth, premolars, and molars with symptomatic irreversible pulpitis or pulp necrosis were selected for the study.

-Standardized endodontic treatment protocol

Treatments were performed by postgraduate endodontic students who presented the same level of training and experience at the time of the study. Anesthesia was administered with 3.6 mL of 2% mepivacaine with 1:100,000 epinephrine (Alphacaine; DFL, Brazil), and cavity access was prepared using 1012 diamond and Endo-Z burs (Dentsply Maillefer, Switzerland) under rubber dam isolation, followed by exploration with a 10 K-file (Dentsply Sirona, Switzerland). The coronal third was flared with Gates Glidden #3 and #2 drills in wide and #2 and #1 in narrow canals. The working length (WL) was determined using an electronic foraminal locator (Novapex; Forum Technologies, Israel) at 1 mm from the apical foramen.

Canal instrumentation used an electric motor (X-smart Plus) (Dentsply-Maillefer, Ballaigues, Switzerland) with rotary or reciprocating systems: Protaper Universal (Dentsply-Maillefer, Ballaigues, Switzerland), Reciproc (VDW, Munich, Germany) or Wave One Gold (Dentsply-Maillefer, Ballaigues, Switzerland). Canal preparation followed manufacturer guidelines, and the file size was determined based on the anatomy of the root canal. Before the chemomechanical preparation, anatomical diameters of the apical foramen and apical constriction were identified through K-FlexoFiles in ascending order to plan and establish similar apical preparation sizes regardless of the tooth group. The main irrigant was 2% CHX gel (Natupharma, Brazil) combined with saline at each instrument change during the instrumentation, a #10 Flex-R file was used to confirm patency. The selection of the instrumentation system was determined by operator convenience.

The smear layer was removed with 5 mL of 17% GA (experimental group) or 17% EDTA (control group), agitated manually for 1 minute with a gutta-percha cone. Irrigation was done with a 30-G needle (NaviTip, Ultradent, USA) 3 mm short of the WL. Final irrigation used 5 mL distilled water, followed by drying with sterile paper tips.

Obturation was performed with gutta-percha cones using the single-cone technique (Odous de Deus, Brazil) and AHPlus cement (Dentsply-Maillefer, Ballaigues, Switzerland). Access cavities were restored with flowable resin (Tetric, Ivoclar Vivadent) and composite resin (Z250, 3M ESPE) using the incremental technique and Single Bond adhesive (3M ESPE). Occlusal adjustments were made. Teeth with iatrogenic issues (perforation, underfillings, or overfillings) were excluded.

Patients were instructed to take 600 mg ibuprofen (Wyeth, Brazil) every 6 hours if needed for pain. If one Tablet did not suffice, subsequent doses were allowed within 6 hours, and the researcher was to be contacted.

-Pain evaluation and consumption of analgesic pills

Pain intensity was analyzed using a VAS scale at 24 hours, 48 hours, and 7 days after endodontic treatment. Patients were trained by a single instructor to use the scale and were contacted by mobile phone and asked, “Are you feeling any discomfort right now? Considering that “0” represents no pain and “10” signifies the worst pain you have ever felt, could you please indicate the level of pain you are currently experiencing on the scale?” The patients sent photographs of the scales to the researcher via a mobile messaging application, and the number of analgesic pills used was also recorded.

-Statistical Analysis

Descriptive and exploratory analyses were conducted. Homogeneity between the groups was assessed using the Student t-test for age variables and the chi-squared or Fisher’s exact test for categorical variables. The nonparametric Mann-Whitney test was used to compare the experimental and control groups for pain scores and analgesic consumption. The non-parametric Friedman and Nemenyi tests were used to compare different time groups. The data were analyzed using IBM SPSS Statistics for Windows, version 25.0 software (IBM Corp, Released 2017: IBM Corp), and the significance level for all analyses was set at 5%.

## Results

**Figure 1:**
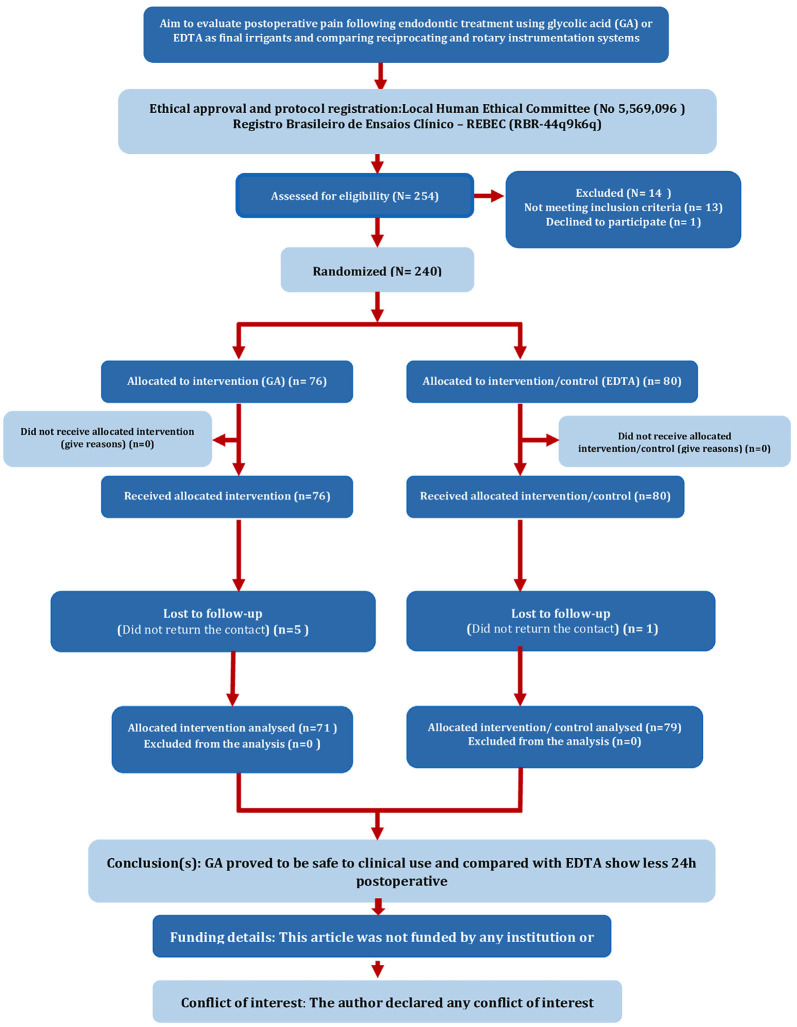
PRIRATE 2020 flow chart.

The study flow chart is shown in Figure 1. The return rate of patients was 82.4% (n=150) within the groups, with 47.4% for GA and 52.6% for EDTA. Of the 150 participants, 91 were female (60.75%) and 59 (39.25%) were male, ranging in age from 18 to 72 years, with a mean age of 42 years. Dropouts simply did not return for follow-up without any specific reason.

The results of the descriptive analysis of demographic and clinical characteristics for each group are presented in  Table 1. Out of the treated patients, 47 (31.4%) were diagnosed with irreversible pulpitis, and 103 (68.6%) had necrotic pulp tissue. Thirty-two (21.3%) of the treated teeth were anterior teeth, 39 (26%) were premolars, and 79 (52.6%) were molars. Preoperative and immediate postoperative radiographs were taken for registration and follow-up.

No statistical differences (*p*>0.05) were found between the group distribution for the following factors: age, sex, tooth position, tooth type, initial crown condition, sensitivity test, palpation test, percussion test, periapical radiographic lesion, initial diagnosis, initial symptomatology, presence of fistula or abscess, and instrumentation technique ( Table 1).

The distribution score and frequency of pain are shown in  Table 2 and  Table 3, respectively. In the 24-hour period, the pain score was significantly lower for GA (*p*<0.05). After this period, pain was similar in both groups (*p*>0.05) ( Table 2). As expected, the frequency of pain decreased over time. Additionally, EDTA showed a 5 to 13% higher frequency in the evaluation periods ( Table 3).

A total of 22 patients (30.9%) in the GA group and 29 (36.7%) in the EDTA group required analgesic intake. The consumption was significantly higher in the first 24 hours compared to 7 days in both groups (*p*<0.05). There was no significant difference between the irrigation solutions (*p*>0.05) ( Table 4).

## Discussion

The present study was designed a randomized prospective double-blind clinical trial to evaluate the endodontic postoperative pain at different time points and analgesic intake in patients treated with two different final irrigation solutions. Among the 150 patients who participated in the present study, postoperative pain occurred in 52% of cases (Table 3), which is consistent with the present by Ehrmann *et al*. (19) and Garcia-Font *et al*. (20). The pain was more frequent in the first 24 hours and decreased subsequently, as shown in the literature (21),(22). Therefore, a mild degree of pain is an expected outcome after endodontic treatment, and the intensity of pain decreases within the first 2 days (23).

Postoperative pain can occur in 3%–58% of patients, and its association of variables such as age, gender, preoperative pain, and the presence of periapical lesions (1). However, in this study, variables like age, gender, tooth position, and periapical condition were not associated with pain ( Table 1). Similar results were reported by Demenech *et al*. (22). Despite this, other studies found an association between pain and increasing age, mandibular teeth (24) and gender, with women being more affected (25).

Clinician’s skills are often measured by the satisfaction with pain control. In the present study, 52% of all cases reported pain within the first 24 hours, and despite falling within the reported range (3-58%), it is considerably high. These findings may be related, in part, to the experience of the operators since the treatments were performed by postgraduate students. Additionally, preoperative pain has been reported to exert a strong impact on postoperative pain levels (1). This factor was reported for 64% of all patients in this study.

In this study, rotary or reciprocating files were used, and no significant effect on pain outcomes was observed. This is in agreement with Comparin *et al*. (21) where both kinematics were similar in terms of incidence, intensity, duration of postoperative pain, and analgesic intake. A study reported that postoperative pain is not influenced by the apical preparation size and taper of the instrument. However, the same authors presented that apical preparation to 2 sizes larger than the initial apical binding file (IABF) with a 4% preparation taper results in a lower success rate compared with preparations done with larger sizes and tapers. Furthermore, the minimum apical preparation size required to adequately disinfect molars was #25/06 or 30/04 in most of the cases (26). In the present study, we determined the anatomical diameter of the apical foramen to establish similar apical preparation sizes regardless of the tooth group. In all treatments, the file size was up to 25/08. To avoid blockage of the apical third, we performed foraminal patency during instrumentation, and despite the potential risk of apical extrusion of infected debris secondary to the mechanical instrumentation beyond the apical foramen that may cause postoperative pain, this procedure has not been associated with higher rates of postoperative pain (27).

Due to its broad antimicrobial effect and low cytotoxicity, which makes it effective in root canal decontamination, chlorhexidine gel was used as an auxiliary chemical substance. Its use is based on literature, which has shown a similar antimicrobial effect to sodium hypochlorite; in addition, it exhibits the unique property of substantivity (28,29).

Different scales have been used to assess postoperative pain (30). The VAS scale has limitations, such as ceiling effects that often leave patients unable to quantify worsening pain (31). Nevertheless, this scale was chosen because of its simplicity and the possibility of scoring with a mobile phone.

GA showed a statistically significantly lower pain level at 24 hours than EDTA, with no difference at 48 hours and 7 days ( Table 2). Therefore, the study hypothesis was partially rejected. This result may be related to the lower cytotoxicity and higher antimicrobial activity demonstrated for GA (12-14). Despite this, it is important to note that if we consider the pain levels on the VAS scale (100 mm), both GA and EDTA are at the mild pain level, thus showing similar results. Dal Bello *et al*. (11) reported that EDTA was cytotoxic even at high dilutions, while GA exhibited cytotoxicity in a dose-dependent manner. In the 24-hour period, the pain score was significantly lower for GA (*p*<0.05). After that, pain was similar in both groups (*p*>0.05) ( Table 2). As expected, the frequency of pain decreased over time. Additionally, EDTA showed a frequency 5 to 13% higher than GA in the evaluation periods ( Table 3). In this study, 17% GA was used. This percentage was chosen based on a previous study (14) and also to match the concentration of EDTA.

No significant difference was found in analgesic intake after endodontic treatment. Furthermore, the results were consistent with more pills being consumed, associated with the presence and intensity of pain. Additionally, no patient consumed analgesic pills on day 7 for GA, which could indicate a lower intensity of pain.

Glycolic acid is a biodegradable acid with potential for use as a final endodontic irrigant in endodontic treatment. In vitro studies have shown its ability to remove the smear layer (11,12), low cytotoxicity, and antimicrobial activity (12-14) and in the present study, the postoperative pain experienced by the patients was similar to or even lower than the levels experienced with the use of EDTA. However, more *in vitro* studies and randomized clinical trials are needed to determine other effects, such as pulp tissue dissolution capacity and long-term effects on dentin and periapical healing.

The research protocol and search blinding were strictly and correctly followed. However, the results of the present study should be interpreted with caution because the treatments were performed by multiple operators, the number of visits to complete the treatment was not considered, and the time taken to complete the treatment was not recorded.

## Conclusions

The present study clinically evaluated glycolic acid as a final rinse solution. This substance proved to be as safe as EDTA for clinical use and showed less 24-hour postoperative pain compared to EDTA in endodontic treatment. No statistically significant difference was observed in 48 and 7 days, and no statistically significant difference was observed in analgesic pill consumption.

## Figures and Tables

**Table 1:** Descriptive Analysis of Demographic and Clinical Characteristics for Each Group (N = 150).

Variable	Measure	Category	Group		p-value
			EDTA	Glycolid Acid
Age (years)	Mean (SD)	-	43.62 (13.91)	40.82 (13.84)	^2 ^>.05
Gender	Frequency (%)	Female	47 (59.5%)	44 (62.0%)	^3^ >.05
		Male	32 (40.5%)	27 (38.0%)	
Tooth position	Frequency (%)	Maxillary arch	43 (54.4%)	45 (63.4%)	^3^ >.05
		Mandibular arch	36 (45.6%)	26 (36.6%)	
Tooth	Frequency (%)	Incisor/canine	19 (24.0%)	13 (18.3%)	^3^ >.05
		Premolar	25 (31.6%)	14 (19.7%)	
		Molar	35 (44.3%)	44 (62.0%)	
Crown initial state	Frequency (%)	Healthy	21 (26.6%)	27 (38.0%)	^3^ >.05
		Restored	58 (73.4%)	44 (62.0%)	
Percussion test	Frequency (%)	Positive	52 (65.8%)	46 (64.8%)	^3^ >.05
		Negative	27 (34.2%)	25 (35.2%)	
Palpation test	Frequency (%)	Positive	32 (40.5%)	21 (29.6%)	^3^ >.05
		Negative	47 (59.5%)	50 (70.4%)	
Vital test	Frequency (%)	Vital	17 (21.5%)	20 (28.2%)	^3^ >.05
		Necrosis	62 (78.5%)	51 (71.8%)	
Periapical lesion	Frequency (%)	Ligament thickening	30 (38.0%)	33 (46.5%)	^3^ >.05
		Periapical lesion	32 (40.5%)	25 (35.2%)	
		Normal	17 (21.5%)	13 (18.3%)	
Diagnosis	Frequency (%)	Irreversible pulpitis	23 (29.1%)	24 (33,8%)	^3^ >.05
		Necrosis	56 (70.9%)	47 (66,2%)	
Initial symptomatology	Frequency (%)	Yes	49 (62.0%)	47 (66.2%)	^3^ >.05
		No	30 (38.0%)	24 (33,8%)	
Fistula	Frequency (%)	Yes	6 (7.8%)	11 (15.5%)	^3^ >.05
		No	73 (92.4%)	60 (84.5%)	
Abcess	Frequency (%)	Yes	2 (2.5%)	5 (7.0%)	^4^ >.05
		No	77 (97.5%)	66 (93.0%)	
Instrumentation technique	Frequency (%)	Rotary	14 (17.7%)	14 (19.7%)	^3^ >.05
		Reciprocanting	65 (82.3%)	57 (80.3%)	

1 Percentage in columns; 2 T student test; 3 Chi-square test; 4 Fisher’s exact test.

**Table 2:** Pain score after 24h, 48h and 7 days.

							^1^p-value
	GA			EDTA			
	Mean (SD)	Interquartile interval	Median (Max and Min)	Mean (SD)	Interquartile interval	Median (Max and Min)	
24 h	1,0 (1.4)	0.0-2.0	0,0 (0.0-6.0) Aa	1.6 (2.1)	0.0-2.0	1.0 (0.0-10.0) Ba	0.0446
48 h	0,5 (0.9)	0.0-1.0	0.0 (0.0-5.0) Bab	1.1 (2,0)	0.0-1.0	0.0 (0.0-10.0) Bb	0.1400
7 days	0.1 (0.3)	0.0-0.0	0.0 (0.0-2.0) Bb	0.4 (1.3)	0.0-0.0	0.0 (0.0-8.0) Bc	0.2581
^2^p-value			0.0001			<0.0001	

Distinct letters (uppercase horizontally and lowercase vertically indicates statistically significant differences) (p≤ .05). 1 Mann Whitney test (*p*< .05). 2 Friedman test (*p*< .05).

**Table 3:** Frequency distribution of the ‘pain’ variable regarding substance and period.

Period	GA		EDTA		Total	
	No pain	Pain	No pain	Pain	No pain	Pain
24 h	39 (54.9%)	32 (45.1%)	33 (41.8%)	46 (58.2%)	72 (48.0%)	78 (52.0%)
48 h	46 (64.8%)	25 (35.2%)	47 (59.5%)	32 (40.5%)	93 (62.0%)	57 (38.0%)
7 days	71 (93%)	5 (7.0%)	69 (87.3%)	10 (12.7%)	135 (90.0%)	15 (10.0%)

**Table 4:** Number of analgesic pills consumption.

							^1^p-value
	GA			EDTA			
	Mean (SD)	Interquartile interval	Median (Max and Min)	Mean (SD)	Interquartile interval	Median (Max and Min)	
24 h	0.5 (0.9)	0.0-1.0	0.0 (0.0-4.0) Aa	0.7 (1.2)	0.0-1.0	0.0 (0.0-5.0) Aa	0.2320
48 h	0.2 (0.5)	0.0-0.0	0.0 (0.0-2.0) Aab	0.4 (0.9)	0.0-0.0	0.0 (0.0-3.0) Aab	0.3177
7 days	0.0 (0.0)	0.0-0.0	0.0 (0.0-0.0) Ab	0.1 (0.6)	0.0-0.0	0.0 (0.0-3.0) Ab	0.2520
^2^p-value			0.0192			0.0087	

Distinct letters (uppercase horizontally and lowercase vertically indicate statistically significant differences) (p≤ .05). 1 Mann Whitney test. 2 Friedman test.

## Data Availability

The datasets used and/or analyzed during the current study are available from the corresponding author.
